# A vitamin E blended highly cross-linked polyethylene acetabular cup results in less wear: 6-year results of a randomized controlled trial in 199 patients

**DOI:** 10.1080/17453674.2020.1807220

**Published:** 2020-08-24

**Authors:** Julie R A Massier, Joost H J Van Erp, Thom E Snijders, Arthur DE Gast

**Affiliations:** a Clinical Orthopedic Research Center – mN, Zeist; b Department of Orthopedic Surgery, Diakonessenhuis, Utrecht, the Netherlands

## Abstract

Background and purpose — Survivorship of total hip arthroplasty (THA) with the ultra-high molecular weight polyethylene (UHMWPE) monoblock cup has been limited due to periprosthetic osteolysis and aseptic loosening, secondary to wear of the UHMWPE. In response, a vitamin E blended highly cross-linked polyethylene (HXLPE) cup was developed. This study set out to compare the wear and clinical 6-year outcomes of vitamin E blended HXLPE with UHMWPE in an isoelastic monoblock cup in patients with hip osteoarthritis who underwent uncemented THA. The 2-year results have been reported previously.

Patients and methods — For this randomized controlled trial 199 patients were included. 102 patients received the vitamin E blended HXLPE uncemented acetabular cup and 97 patients the uncemented UHMWPE monoblock cup. Clinical and radiographic parameters were obtained preoperatively, directly postoperatively, and at 3, 12, 24, and 72 months. Wear rates were compared using the femoral head penetration (FHP) rate.

Results — 173 patients (87%) completed the 6-year follow-up. The mean NRS scores for rest pain, load pain, and patient satisfaction were 0.3 (SD 1), 0.6 (SD 1), and 8.6 (SD 1) respectively. The mean Harris Hip Score was 93 (SD 12). The FHP rate was lower in the vitamin E blended HXLPE cup (0.028 mm/year) compared with the UHMWPE cup (0.035 mm/year) (p = 0.002). No adverse reactions associated with the clinical application of vitamin E blended HXLPE were observed. 15 complications occurred, equally distributed between the two cups. The 6-year survival to revision rate was 98% for both cups. There was no aseptic loosening.

Interpretation — This study shows the superior performance of the HXLPE blended with vitamin E acetabular cup with clinical and radiographic results similar to the UHMWPE acetabular cup.

The use of HXLPE in monoblock cups used for total hip arthroplasty (THA) has been shown to reduce wear significantly (Muratoglu et al. 2001, Martell et al. 2003, Devane et al. 2017). However, the cross-linking of polyethylene forms free radicals, which leads to a decrease of long-term oxidative stability, causing embrittlement of the HXLPE (Sutula et al. 1995).

In order to prevent this embrittlement, a new generation of HXLPE has been developed in which the polyethylene is stabilized with vitamin E. Vitamin E reacts with free radicals, protecting against oxidative degradation, avoiding long-term oxidation, embrittlement, and mechanical failure (Oral et al. 2004). Several studies have shown good in vitro and vivo results of the vitamin E stabilized HXLPE in terms of wear rates and mechanical properties (Oral et al. 2006, Halma et al. 2015, Nebergall et al. 2017, Scemama et al. 2017, Lambert et al. 2018, Snijders et al. 2019, van Erp et al. 2020). However, mid-term results of large randomized controlled trials (RCT) corroborating these findings have not been published.

The mechanical characteristics of the monoblock acetabular cup potentially reduce polyethylene wear and acetabular osteolysis. Several studies have shown promising survival rates and reduced wear and osteolysis frequencies (Young et al. 2002, Gwynne-Jones et al. 2009, Pakvis et al. 2011).

Survivorship of total hip arthroplasty (THA) with an ultra-high molecular weight polyethylene (UHMWPE) monoblock cup has been limited due to periprosthetic osteolysis and aseptic loosening, secondary to wear of the UHMWPE (Harris 2001). The number of patients in need of a THA will increase in the future and these patients will have higher expectations regarding performance and durability. Therefore, highly cross-linked polyethylene (HXLPE) was introduced in the late 1990s (Kurtz et al. 2011).

This RCT compares the wear rates expressed as femoral head penetration (FHP) between the uncemented vitamin E blended HXLPE acetabular cup and the conventional UHMWPE acetabular cup after 6 years. Secondary outcomes are the effect of an increased head size on the wear, clinical performance, and complication rates between the two cups.  

## Patients and methods

### Study design

This RCT was carried out at the Diakonessenhuis Hospital Utrecht/Zeist, a medium-sized general hospital in the Netherlands. Between 2011 and 2014, 199 patients were included, and randomly allocated in 2 groups (van Erp et al. 2020). Description of patient inclusion and exclusion criteria, baseline characteristics, surgical procedures, and implants are described in the 2-year follow-up of this RCT by van Erp et al. (2020). After enrollment, baseline characteristics, Harris Hip Score (HHS), and Numeric Rating Scale (NRS) score for patient satisfaction, rest, and load pain were documented.

After randomization, 102 patients received an uncemented vitamin E blended HXLPE cup (RM uncemented monoblock Pressfit Vitamys cup, Mathys Ltd, Bettlach, Switzerland) and 97 patients received a conventional UHMWPE cup (RM uncemented monoblock Pressfit, Mathys Ltd, Bettlach, Switzerland).

Patients were scheduled for clinical and radiological follow-up on the first day postoperatively, and at 3, 12, 24, and 72 months. At each follow-up HHS and NRS scores, as well as complications, were documented and anteroposterior radiographs (AP) of the pelvis in supine position were taken. The primary outcome of this study was the linear FHP rate. Secondary outcomes were the clinical outcomes and the effect of increased head size.

Follow-up was performed by 1 independent investigator (JE) who was blinded to the intervention. In order to reduce bias, the patient was blinded until completion of 6-year follow-up and the surgeon did not perform the follow-up measurements. Subjects were able to leave the study at any time or for any reason, without any consequences. The investigators were able to withdraw a subject from the study for urgent medical reasons. There was no replacement of subjects after withdrawal. For the sample size calculation see van Erp et al. (2020).

### Procedure

Critical aspects of the surgical procedure were standardized for both groups. Surgeons required knowledge of the allocated treatment. Therefore, they were not blinded to the intervention, and were informed about the cup type during surgery. 7 orthopedic surgeons with vast experience in uncemented THA performed the procedures. Alumina ceramic femoral prosthetic heads (BIONIT2, Mathys Ltd, Bettlach, Switzerland) of 28, 32, or 36 mm were used and an uncemented hydroxyapatite coated stem (Twinsys, Mathys Ltd, Bettlach, Switzerland) was implanted.

Pre- and postoperative care were protocolled in order to ensure similar perioperative regimens. All patients received cefazolin prophylaxis during 24 hours perioperatively and thromboprophylaxis with low molecular weight heparin for 6 weeks postoperatively. Patients followed the same rehabilitation regimen, starting on the first day after surgery.

### Radiological assessment

Radiological assessment was performed by 1 of the authors (JM) according to a standardized form; see van Erp et al. (2020).

### Statistics

Statistical analysis was performed using SPSS Statistics, version 23.0 (IBM Corp, Armonk, NY, USA). The distribution of the data was checked using the Kolmogorov–Smirnov test. A Pearson chi-square test was used to test for differences between groups in surgical approach, head size, and radiographic specifics (Brooker classification, radiographic lucency around the cup and stem, and osteolysis around the stem).

A paired t-test was used to test for significant differences in both groups between the FHP rate in the first year and in the last 5 years to determine the steady state. The Mann–Whitney U-test was used to test for significant differences between groups for the FHP rate at 6 years.

### Ethics, registration, funding, data sharing, and potential conflicts of interest

The procedures performed in this study, involving human participants, were in accordance with the ethical standards of the institutional and/or national research committee, with the 1964 Declaration of Helsinki and its later amendments or comparable ethical standard and the CONSORT statement. All patients gave informed written consent. The protocol was approved by the local institutional review board and registered at the Central Commission Human-Related research (CCMO) Registry as HipVit trial (NL 32832.100.10, R-10.17D/HIPVIT 1).

This study was funded by the Clinical Orthopedic Research Center – mN. Data are available from the corresponding author on reasonable request. The authors have no competing interests.

## Results

**Figure 1.**
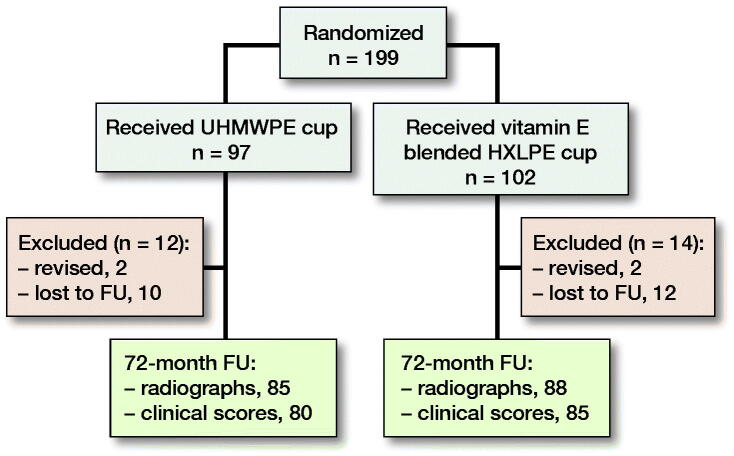
Summary of follow-up.

177 patients (89%) completed 6-year follow-up. The mean time to final follow-up was 70 months (SD 9). Patient characteristics and surgery specifics are displayed for both groups (Tables 1 and 2). 22 patients (11%) were lost to follow-up (Figure 1). 6 patients died due to other diseases, 12 patients were unreachable, and 4 patients left the study because of severe comorbidity.

**Table 3.** Femoral head penetration rates in mm. Values are mean (SD) and [95% confidence intervals]

Follow-up interval (year)	Vitamin E blended HXLPE	UHMWPE	p-value
Penetration (mm)			
0–1	0.24 (0.14) [0.21–0.27]	0.25 (0.13) [0.22–0.27]	0.9
0–2	0.27 (0.14) [0.25–0.30]	0.28 (0.12) [0.26–0.31]	0.8
0–6	0.38 (0.15) [0.35–0.41]	0.44 (0.16) [0.40–0.47]	0.01
1–6	0.14 (0.07) [0.13–0.16]	0.18 (0.08) [0.16–0.19]	0.002
2–6	0.07 (0.05) [0.06–0.08]	0.09 (0.06) [0.08–0.10]	0.05
Penetration rates (mm/year) at			
2 years	0.046 (0.030) [0.040–0.052]	0.056 (0.038 [0.048–0.065]	0.05
6 years	0.028 (0.013) [0.026–0.031]	0.035 (0.016 [0.032–0.039]	0.002

For abbreviations, see Table 1.

**Table 2.** Surgery specifics. Values are n (%)

		Vitamin E	
	Total	blended HXLPE	UHMWPE
	n = 199	n = 102	n = 97
Approach			
Direct lateral	98 (49)	45 (44)	53 (55)
Posterolateral	85 (43)	46 (45)	39 (40)
Anterolateral	16 (8)	11 (11)	5 (5)
Head size			
28 mm	22 (11)	2 (2)	20 (21)
32 mm	112 (56)	35 (34)	77 (79)
36 mm	65 (33)	65 (64)	NA
Inclination			
< 35°	16 (8)	5 (5)	11 (11)
35–40°	47 (24)	20 (19)	27 (28)
41–45°	54 (27)	26 (26)	28 (29)
46–50°	51 (26)	31 (30)	20 (21)
> 50°	31 (16)	20 (20)	11 (11)

For abbreviations, see Table 1.

**Table 1.** Demographics. Values are n (%) unless otherwise specified

		Vitamin E	
	Total	blended HXLPE	UHMWPE
	n = 199	n = 102	n = 97
Age, mean (SD)	65 (5)	66 (5)	65 (5)
Weight (kg), mean (SD)	79 (13)	78 (13)	79 (14)
Female	141 (71)	77 (75)	64 (66)
Diagnosis			
Primary osteoarthritis	191 (96)	98 (96)	93 (96)
Secondary osteoarthritis	2 (1)	1 (1)	1 (1)
Inflammatory arthritis	1 (1)		1 (1)
Femoral head			
avascular necrosis	1 (1)		1 (1)
Hip dysplasia	4 (2)	3 (3)	1 (1)
Vitamin E blended HXLPE: vitamin E diffused highly cross-linked polyethylene.			
UHMWPE: ultra-high molecular weight polyethylene.			
Table 6. Postoperative complications after 6-year follow-up			
	Vitamin E		
Total	blended HXLPE	UHMWPE	
Complication	n = 199	n = 102	n = 97
Malposition cup	1	1	–
Pulmonary embolism	1	1	–
Deep venous thrombosis	1	1	–
Neurological damage	2	–	2
Superficial wound infection	2	1	1
Deep infection	1	1	–
Leg length discrepancy	1	1	–
Dislocation	6	2	4
Total	15 (8%)	8 (8%)	7 (7%)

4 patients (2%) were excluded because they underwent revision surgery. In the vitamin E blended HXLPE group 2 patients underwent revision surgery: 1 because of infection and 1 for recurrent instability. In the UHMWPE group, 2 patients underwent revision surgery: 1 for cup malpositioning and 1 for recurrent instability. Furthermore, clinical data were missing for 5 patients in the UHMWPE and 3 in the vitamin E blended HXLPE groups.

The head sizes used in the vitamin E blended HXLPE group were larger than the head sizes in the control group (p < 0.001). This is mainly because the 36-mm head size is not available for the UHMWPE cup. Bivariate analysis showed similar rates of heterotopic ossifications, radiographic lucencies around the cup and lucencies and osteolysis around the stem.

### Femoral head penetration

**Figure 2.**
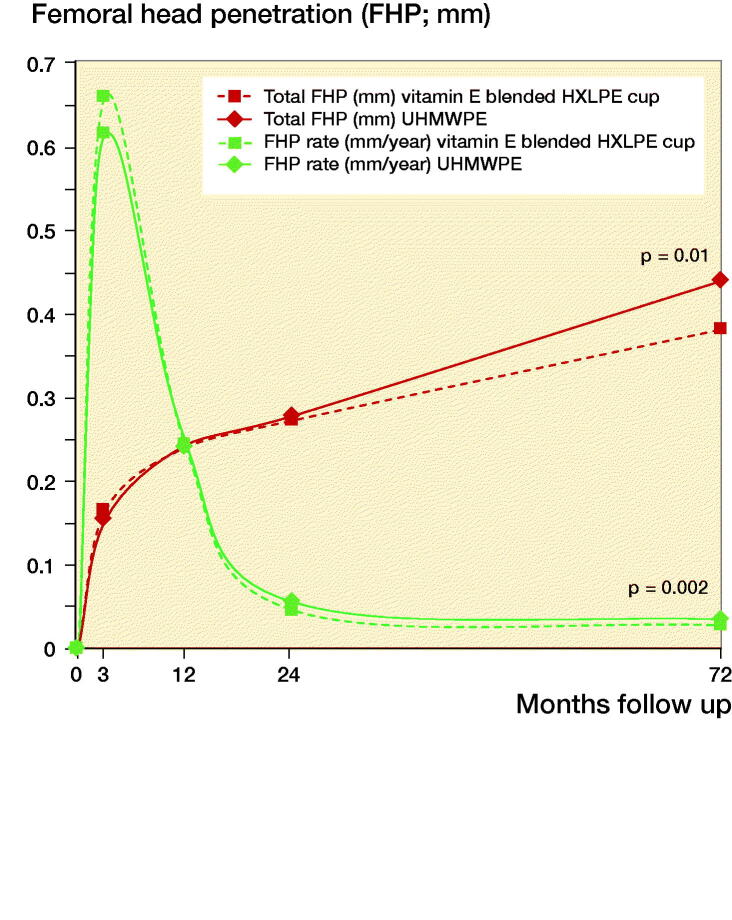
Total femoral head penetration (FHP) in mm and mean FHP rate in mm/year.

The FHP rates were normally distributed, except for the FHP rate from 1 to 6 years. The total FHP after 6 years was 0.38 mm in the vitamin E blended HXLPE cup and 0.44 mm in the UHMWPE cup (p = 0.01) (Table 3). The mean FHP rate from 1 to 6 years, thus excluding the bedding-in time of the first year, was 0.028 and 0.035 mm/year for the vitamin E blended HXLPE cup and UHMWPE cup respectively (Figure 2). The mean FHP rate of the vitamin E blended HXLPE cup after 6 years was lower compared with the FHP rate of the UHMWPE cup (p = 0.002) (Table 3). FHP rates (in mm/year) for each head size in both groups are shown in Table 4 (see Supplementary data). No significant differences in wear between head sizes of the same cup type were found.

A sub-analysis of the 28- and 32-mm head sizes was performed. No statistically significant difference in FHP rates between the UHMWPE (0.035 mm/year) and vitamin E blended HXLPE cup (0.031 mm/year) were found at 6-year follow-up (p = 0.2).

There was a statistically significant difference between the FHP rate in the second year and in the last 5 years in both groups (vitamin E blended HXLPE cup; p < 0.001, UHMWPE cup; p < 0.001).

### Clinical outcomes

**Figure 3.**
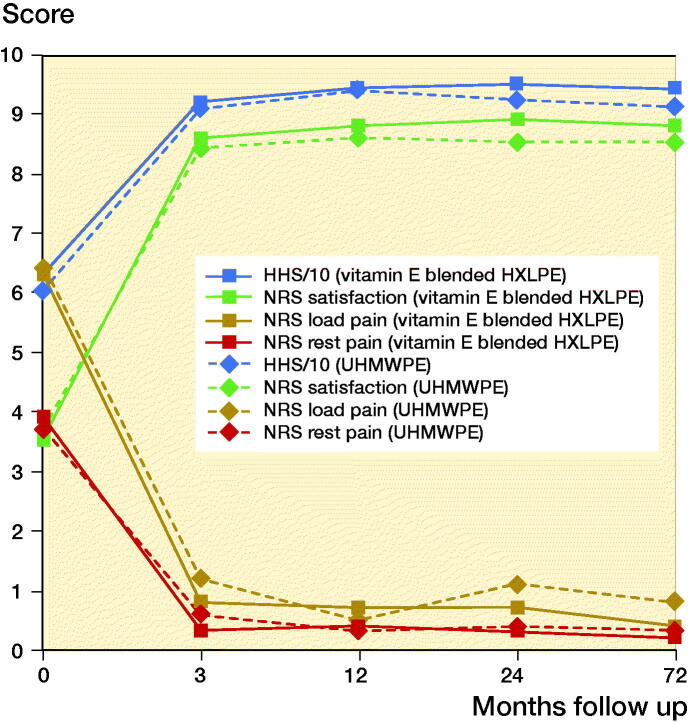
Numeric Rating Scale and Harris Hip Score.

NRS and HHS after 6 years were similar for both groups (Figure 3, Table 5, see Supplementary data). The all-cause 6-year survival to revision was 98% for both groups. The 6-year survival rate for aseptic loosening was 100% in both groups.

### Complications

No adverse reactions associated with the clinical application of vitamin E-blended HXLPE were observed during this study. In 2 patients with a vitamin E blended HXLPE cup, a perioperative fracture of the femur occurred. In 1 patient with a UHMWPE cup, a postoperative fracture of the acetabulum occurred. All patients were treated nonoperatively and recovered completely without further complications.

Within 6-year follow-up 15 complications occurred, among which were 6 patients with dislocations (3%) (Table 6). In the vitamin E blended HXLPE group 2 dislocations occurred and in the UHMWPE 4 (p = 0.4). All patients recovered completely, except for 2 patients who still had mild paresthesia caused by neuropraxia of the sciatic nerve.  

## Discussion

This large, mid-term RCT compares 2 similar acetabular monoblock cups: a vitamin E blended HXLPE and a UHMWPE cup. The vitamin E blended HXLPE cup showed a statistically significant different 2-dimensional linear FHP rate of 0.028 mm/year compared with the UHMWPE cup (0.035 mm/year) (p = 0.002). Good mid-term clinical and radiological outcomes of both cups are evident. No adverse reactions or abnormal mechanical behavior concerning the vitamin E additive were observed. No signs of osteolysis or aseptic loosening were observed in either group.

Many studies have shown a substantial amount of the FHP occurring within the first year after surgery, due to bedding-in or creep of the polyethylene (Sychterz et al. 1999, McCalden et al. 2005). Therefore, the first year of linear FHP was attributed to creep (Rochcongar et al. 2018, Snijders et al. 2019). The total FHP in this study was 0.38 mm in the vitamin E blended HXLPE cup and 0.44 mm in the UHMWPE cup (p = 0.01). This is well within the range of results reported in current literature (Dorr et al. 2005, Lambert et al. 2018, Snijders et al. 2019). After bedding-in, steady-state penetration rate is reached. This study showed a statistically significant difference between the FHP rate in the first and second year of follow-up in both groups (van Erp et al. 2020). However, there was no significant difference between the FHP rate in the second year and last 4 years in either group, suggesting steady-state penetration with a constant wear rate has been reached (Figure 2).

Several studies have shown superior performance of vitamin E addition to HXLPE for protection from oxidation, preserving mechanical properties, reduced wear debris, and bacterial adhesion compared withthe UHMWPE cups. This suggests that adding vitamin E may prevent osteolysis, implant loosening, and eventually revision surgery (Scemama et al. 2017, Yamamoto et al. 2017, Galea et al. 2018, Rochcongar et al. 2018). Shareghi et al. (2017) found in 37 vitamin E-infused HXLPE cups an FHP rate of 0.04 mm/year and in 26 HXLPE cups an FHP rate of 0.07 mm/year between 2 and 5 years, using radiostereometric analysis (RSA). These slightly higher wear rates could be explained by the shorter period of 5 years’ follow-up and a different measuring technique. A small RCT comparing the same cups in 62 patients found a statistically significant difference (p < 0.001) between the steady-state FHP rate in a vitamin E blended HXLPE cup (0.020 mm/year) and a UHMWPE cup (0.058 mm/year) after 3 years of follow-up, using radiostereometric analysis (RSA) (Rochcongar et al. 2018). These results are comparable to our findings, only with a smaller patient cohort and shorter follow-up time. Furthermore, Dumbleton et al. (2002) showed osteolysis and increased risk of revision surgery due to loosening are rare below a linear wear rate of 0.1 mm/year. Our study demonstrated no osteolysis or revisions for aseptic loosening in either group after 6 years of follow-up.

This is the first blinded RCT, using a reliable measuring technique, with substantially more patients and a similar control group to compare the effect of vitamin E on wear rates of 2 similar cups. A high follow-up rate (89%) has been reached. Our results are with a similar order of magnitude in FHP rates compared with current literature (Halma et al. 2015, Snijders et al. 2019).

This study has several limitations. First, patients in the vitamin E blended HXLPE group received significantly larger head sizes; however, multivariate analysis showed no statistically significant effect of head size on the wear rates in either cup. Besides, in the literature, the effect of a greater head size on the polyethylene wear rates is disputed (Lachiewicz et al. 2009, 2016, van Erp et al. 2020). Furthermore, due to the availability of the vitamin E blended HXLPE cup on the Dutch market, surgeons only have a choice of larger head sizes in most similar cup sizes. In its manufacturing process thinner polyethylene liners are applied because less wear is expected. The possibility to employ larger heads in the same patient due to a thinner polyethylene liner is an advantage of the RM Pressfit vitamys cup (vitamin E blended HXLPE cup) over the UHMWPE cup, since it results in lower risk of dislocation (Knahr 2012). In our study we saw fewer dislocations in the vitamin E blended HXLPE group compared with the UHMWPE group (2 versus 4). Second, despite our follow-up rate of 89%, the loss of 11% is a limitation as the unreachable patients could have been admitted to other hospitals. This should be taken into account while reviewing the results. Third, only a linear, 2-dimensional penetration measurement was performed, using the View Pro-X software instead of the 3D RSA measuring method (Callary et al. 2015). RSA requires the use of tantalum beads placed into the implants and bone. This limits the use of RSA (Lawrie et al. 2003). The View Pro-X software was found to be a reliable method in clinical practice for the assessment of linear FHP to determine the polyethylene wear, with good inter- and intra-class reliability (Martell et al. 2003, Geerdink et al. 2008). Using this software allows a non-invasive, precise measuring method for large patient cohorts.

In conclusion, this large RCT shows the excellent and superior mid-term performance of the vitamin E blended HXLPE cup, with significantly less wear in terms of FHP rates compared with the UHMWPE cup. There was no difference between the two groups in terms of clinical outcomes, revision rates, and complications. Since differences in wear rates are few, it is hard to say whether clinical differences will be found in future follow-up. Further long-term follow-up is needed to assess whether these lower wear rates in vitamin E blended HXLPE cups result in less osteolysis and aseptic loosening, compared with conventional UHMWPE.

## Supplementary Material

Supplemental Material

## Supplementary data

Tables 4 and 5 are available as supplementary data in the online version of this article, http://dx.doi.org/10.1080/17453674. 2020.1807220
