# Supplementary material for: A vitamin E blended highly cross-linked polyethylene acetabular cup results in less wear: 6-year results of a randomized controlled trial in 199 patients
Source: Acta Orthop. 2020 Aug 24;91(6):705–10. doi: 10.1080/17453674.2020.1807220 (PMC8023918; doi:10.1080/17453674.2020.1807220)
Supplement: Supplemental Material [file IORT_A_1807220_SM8840.pdf]

Supplementary data

Table 4. Femoral head penetration rate (mm/year) per head size after 6-year follow-up. Values are mean (SD)

Head size	Vitamin E blended HXLPE	UHMWPE
28 mm	0.023 (0.018)	0.033 (0.014)
32 mm	0.030 (0.013) [a]	0.036 (0.017)
36 mm	0.027 (0.013) [a]	

[a] 32 mm vs. 36 mm: p = 0.01.

Table 5. Numeric Rating Scale (NRS) and Harris Hip Scores (HHS) after 6-year follow-up. Values are mean (SD)

Category	Total n = 163	Vitamin E blended HXLPE n = 85	UHMWPE n = 80	p-value
NRS rest pain	0.3 (1)	0.2 (1)	0.3 (1)	0.7
NRS load pain	0.6 (1)	0.4 (1)	0.8 (2)	0.07
NRS satisfaction	8.6 (1)	8.8 (1)	8.5 (1)	0.06
HHS/10	9.3 (1)	9.4 (1)	9.1 (1)	0.09
